# Estimating Loss to Follow-Up in HIV-Infected Patients on Antiretroviral Therapy: The Effect of the Competing Risk of Death in Zambia and Switzerland

**DOI:** 10.1371/journal.pone.0027919

**Published:** 2011-12-19

**Authors:** Franziska Schöni-Affolter, Olivia Keiser, Albert Mwango, Jeffrey Stringer, Bruno Ledergerber, Lloyd Mulenga, Heiner C. Bucher, Andrew O. Westfall, Alexandra Calmy, Andrew Boulle, Namwinga Chintu, Matthias Egger, Benjamin H. Chi

**Affiliations:** 1 Division of International and Environmental Health, Institute of Social and Preventive Medicine (ISPM), University of Bern, Bern, Switzerland; 2 Data Centre, Swiss HIV Cohort Study, University Hospital Lausanne, Lausanne, Switzerland; 3 Zambian Ministry of Health, Lusaka, Zambia; 4 Centre for Infectious Disease Research in Zambia (CIDRZ), Lusaka, Zambia; 5 Schools of Medicine and Public Health, University of Alabama, Birmingham, Alabama, United States of America; 6 Division of Infectious Diseases and Hospital Epidemiology, University Hospital Zurich, University of Zurich, Zurich, Switzerland; 7 Basel Institute for Clinical Epidemiology and Biostatistics, University Hospital Basel, Basel, Switzerland; 8 HIV/Aids Unit, Division of Infectious Diseases, Department of Internal Medicine, University Hospital Geneva, Geneva, Switzerland; 9 School of Public Health and Family Medicine, University of Cape Town, Cape Town, South Africa; University of Cape Town, South Africa

## Abstract

**Background:**

Loss to follow-up (LTFU) is common in antiretroviral therapy (ART) programmes. Mortality is a competing risk (CR) for LTFU; however, it is often overlooked in cohort analyses. We examined how the CR of death affected LTFU estimates in Zambia and Switzerland.

**Methods and Findings:**

HIV-infected patients aged ≥18 years who started ART 2004–2008 in observational cohorts in Zambia and Switzerland were included. We compared standard Kaplan-Meier curves with CR cumulative incidence. We calculated hazard ratios for LTFU across CD4 cell count strata using cause-specific Cox models, or Fine and Gray subdistribution models, adjusting for age, gender, body mass index and clinical stage. 89,339 patients from Zambia and 1,860 patients from Switzerland were included. 12,237 patients (13.7%) in Zambia and 129 patients (6.9%) in Switzerland were LTFU and 8,498 (9.5%) and 29 patients (1.6%), respectively, died. In Zambia, the probability of LTFU was overestimated in Kaplan-Meier curves: estimates at 3.5 years were 29.3% for patients starting ART with CD4 cells <100 cells/µl and 15.4% among patients starting with ≥350 cells/µL. The estimates from CR cumulative incidence were 22.9% and 13.6%, respectively. Little difference was found between naïve and CR analyses in Switzerland since only few patients died. The results from Cox and Fine and Gray models were similar: in Zambia the risk of loss to follow-up and death increased with decreasing CD4 counts at the start of ART, whereas in Switzerland there was a trend in the opposite direction, with patients with higher CD4 cell counts more likely to be lost to follow-up.

**Conclusions:**

In ART programmes in low-income settings the competing risk of death can substantially bias standard analyses of LTFU. The CD4 cell count and other prognostic factors may be differentially associated with LTFU in low-income and high-income settings.

## Introduction

In most settings, regular attendance at health facilities is critical to the comprehensive medical care of HIV-infected patients. During these visits, health care workers examine patients for clinical progression, provide and monitor combination antiretroviral therapy (ART), and counsel them on minimizing the risk of HIV transmission. Despite this need for regular monitoring, significant loss to follow-up has been demonstrated in ART programmes and cohort studies – both in industrialized and resource-limited settings – at rates often exceeding reported mortality [1]. In a collaborative analysis of patients who had been on ART for six months in Europe and North America, 27% were lost to follow-up over a median of 3.75 years [2]. In the Swiss HIV Cohort Study (SHCS) loss to follow-up was 121 per 1000 patient years in the pre ART era [3], but decreased in the ART era to 33.6 per 1000 patient years [4]. In industrialized settings less advanced disease, younger age, injection drug use and homelessness have been associated with loss to follow-up [5]–[7].

Loss to follow-up is more common in resource-poor settings. In an Antiretroviral Treatment in Lower Income Countries (ART-LINC) study, loss to follow-up after 1 year was above 40% in some programs [8], and associated with more advanced clinical disease and lower CD4 cell counts. Similarly, an analysis of the large ART programme jointly administered by the Zambian Ministry of Health and the Centre for Infectious Disease Research in Zambia (CIDRZ) showed that predictors of treatment failure or death also predicted loss to follow-up [9]. A meta-analysis of studies that traced patients lost to follow-up to ascertain their vital status showed that in sub-Saharan Africa 40% of those traced had died [10]. High rates of loss to follow-up may affect mortality estimates in ART programmes if patients lost to follow-up have a different prognosis compared to similar patients remaining in care [11]. Obtaining valid estimates of loss to follow-up at different points in time is therefore important when evaluating ART programmes.

Death is a competing risk of loss to follow-up: patients who die can no longer become lost to follow-up. Competing risks are defined as events that prevent the outcome of interest from occurring. They are common in longitudinal studies and are particularly important in populations at high risk of death [12], [13]. For example, death from all causes is a competing risk when studying recurrences after treatment of cancer, and death from other causes is a competing risk when studying a specific cause of death. In standard Kaplan-Meier analyses, the follow-up of those developing a competing event is simply censored, assuming that the probability of the outcome of interest is the same as that of comparable patients remaining under observation. However, this assumption is invalid because the outcome of interest can no longer occur in those developing the competing event, and such analyses will therefore overestimate the probability of the outcome of interest. This situation can be seen as an extreme form of ‘informative’ censoring, where censoring is associated with the probability of the outcome [14]. Analyses that ignore competing events are however regularly published [7], [15], [16] even though they may produce misleading results.

We examined how the competing risk of death affected estimates of loss to follow-up in cohorts of patients starting ART in Zambia and Switzerland.

## Methods

We analysed data from two well-described HIV cohorts: a Zambian cohort supported by the Centre for Infectious Disease Research in Zambia (CIDRZ) and the Zambian Ministry of Health [9], [17] (referred to hereafter as the CIDRZ cohort) and the Swiss HIV Cohort Study [4].

### CIDRZ cohort, Zambia

CIDRZ-supported activities in HIV care and treatment began in 2004 across four sites in Lusaka. Since then, the programme has expanded to 68 facilities, most of them government health centres and hospitals. Across all sites, clinical care is standardized according to the Zambian National HIV guidelines [18]. Over the analysis period, individuals were eligible for ART when: (a) they were diagnosed with a stage IV conditions according to World Health Organization (WHO) criteria; (b) their CD4 cell count was below 200 cells/µL; or (c) they had a stage III condition and their CD4 was between 200 and 350 cells/µL. Clinical and immunologic monitoring occurred every 3–6 months. Although viral load testing is available, its use is limited for reasons of cost and operational constraints [19], particularly outside of Lusaka. Patients with missed visits are contacted by community health workers and reminded about their appointments [20]. All patient-level data are entered into a comprehensive electronic medical record supported by the Ministry of Health. Additional details of the CIDRZ programme can be found elsewhere [9], [17]. Approval for use of these programmatic data was obtained from the University of Zambia Research Ethics Committee (Lusaka, Zambia) and the University of Alabama at Birmingham (Birmingham, AL, USA). Only routine clinical data were analyzed for the present study and informed consent from patients was not obtained.

### Swiss HIV Cohort Study, Switzerland

Established in 1988, the Swiss HIV Cohort Study (SHCS) is a national prospective cohort study of HIV-infected patients followed up at outpatient departments of five University hospitals (Basel, Berne, Geneva, Lausanne and Zurich) and two Cantonal hospitals (Lugano and St. Gallen). A comparison with official AIDS notifications and deaths indicated that about 70% of all patients living with AIDS in Switzerland participate in the study [21]. Data collection and study procedures are standardised. Detailed information on demographics, mode of HIV acquisition, risk behaviours, clinical events, laboratory results (i.e. viral load, CD4 cell counts and additional data), and treatments is collected at registration and then at 6-monthly intervals. Clinical AIDS diagnoses (Center for Disease Control and Prevention [CDC] stage C) are recorded by the treating physician on the basis of the revised 2008 CDC criteria [22]. All services including ART and laboratory testing are covered by compulsory health insurance. Patients who do not attend the next follow-up visit are actively traced. Further details about the SHCS programme is provided elsewhere [4]. Local ethics committees at all seven SHCS sites approved the study and written informed consent was obtained from all participants.

### Eligibility criteria and definitions

Treatment-naïve patients aged 18 years or older who started ART between 2004 and 2008 were eligible for the present analysis. ART was defined as a combination of at least three antiretroviral drugs from at least two classes. The clinical stage of disease was classified as less advanced (CDC stage A/B, WHO stage I/II) or advanced (CDC stage C, WHO stage III/IV). Patients were grouped according to their CD4 cell count (<100; 100–199; 200–349; ≥350 cells/µL) and body mass index (BMI; <20; 20–25; >25 kg/m^2^) at the start of ART.

### Outcomes

Two endpoints were considered: loss-to follow up, and death as a competing event. Patients were considered lost to follow-up if they were not seen for more than 14 months, irrespective of whether or not they came back later on. These patients were censored at their last visit, or the last visit before the gap in follow-up. In Zambia, empirical work suggested that the optimal definition for loss to follow-up was 60 days since the last missed clinic appointment [23]; however, because this was not appropriate for the Swiss cohort, we used 14 months as the threshold for both cohorts. Patients who were transferred out to another site were also censored at their last visit. We used an “intent-to-continue-treatment” approach and thus ignored subsequent changes to treatment, including interruptions and terminations.

### Statistical analyses

We first compared the ‘naïve’ Kaplan-Meier analyses to competing risk cumulative incidence curves using the Aalen-Johansen estimator [24]. Individuals contributed person-time from the start of ART until the last follow-up visit or known date of death. We then compared cause-specific hazard ratios from Cox models across baseline CD4 groups with the subdistribution hazard ratios from the model described by Fine and Gray [13], [25]–[27]. In the Cox model observations with failures from other causes (death or loss to follow-up respectively in this study) are censored. The Cox model is a reasonable choice but is restricted to modelling instantaneous risk functions [28]. The Fine and Gray model makes use of the subdistribution hazard to model cumulative incidence and thus quantify the overall benefit or harm of an exposure [28]. Patients with the competing event were kept at risk and continued to contribute person time, with the remaining time at risk weighted by the inverse probability of censoring [25]. Analyses were adjusted for age, sex and categories of CD4 cell count, BMI, and clinical stage at start of ART. In CIDRZ weight and height was sometimes not measured on the day of starting ART and therefore the last value before starting ART was carried forward. Partial residual analysis and model-based diagnostics were used to test the assumption of proportional hazards in all models. Data were analysed using SAS version 9.2 (SAS Institute Inc., Cary, NC, USA) and R version 2.13.1 (www.r-project.org).

## Results

### Patient characteristics

In the CIDRZ cohort, 89,339 patients were followed for a total of 124,163 person-years. The median observation time was 10.0 months (interquartile range [IQR] 1.8–23.4 months). All patients were presumed to have been infected through heterosexual contacts, although these data are not routinely collected. The number of patients starting ART increased rapidly from 5,667 in 2004 to 25,772 in 2008. A median of 1,188 patients (range: 139–2,173 patients) started ART at each site and 6,988 patients (7.8%) were known to have been transferred to another facility.

In the SHCS 1,860 patients were followed for 3,989 person-years, including 755 patients (40.6%) infected through sex between men, 852 patients (45.8%) infected heterosexually, 196 patients (10.5%) infected through intravenous drug use and 57 patients (3.1%) infected through an unknown source. The median observation time was 33.7 months (IQR 24.1–39.0). The number of patients starting ART remained stable over time with a range of 372 to 439 patients starting each year. The median number of patients per site was 230 (range 45–725) and 19 (1.02%) were transferred out to another site.

Table 1 shows the characteristics of the patients at start of ART. The patients in CIDRZ were younger, more likely to be female, and more likely to present at an advanced disease stage. The most commonly prescribed ART regimens varied substantially between the two cohorts. In the SHCS 19.7% of patients used a protease inhibitor-based regimen (mostly lamivudine [3TC], zidovudine [AZT], and lopinavir [LPV]). In Zambia, most patients started ART with a NNRTI-based regimen: 41.7% initiated a combination of stavudine (d4T), 3TC, and nevirapine (NVP), while 21.1% initiated a combination of AZT, 3TC, and NVP.

**Table 1:** Baseline characteristics and mortality at end of follow-up among patients starting antiretroviral therapy in two cohorts in Zambia and Switzerland.

	Zambia (CIDRZ)			Switzerland (SHCS)		
	N	Median or %	IQR	N	Median or %	IQR
**Total No. of patients**	89,339	100%		1,860	100%	
**CD4 cell count (cells/µL)**	82,955	147	74–240	1,860	322	164–491
**Age (years)**	89,339	35	30–42	1,860	38	32–45
**Body mass index (kg/m^2^)**	89,233	19.7	17.7–22.1	1,860	23.6	21.5–26.1
**Sex**						
Women	54,432	60.9%		592	31.8%	
Men	34,907	39.1%		1,268	68.2%	
**Advanced clinical stage** *						
Women	35,476	65.1%		115	30.1%	
Men	25,821	73.9%		477	32.2%	
**Most common treatment regimens**						
d4T/3TC, NVP	37,254	41.7%				
AZT/3TC, NVP	18,850	21.1%				
AZT/3TC, EFV				166	8.9%	
ETC, TNV, EFV	12,060	13.5%				
AZT/3TC, LPV				367	19.7%	
ETC, TNV, LPV				190	10.2%	
**Mortality**						
Women	4,512	8.3%		10	1.7%	
Men	3,986	11.5%		29	2.2%	

CIDRZ: Centre for Infectious Disease Research in Zambia; SHCS: Swiss HIV Cohort Study; IQR: interquartile range; d4T: stavudine; 3TC: lamivudine; NVP: nevirapine; AZT: zidovudine; EFV: efavirenz; ETC: emtricitabine; TNV: tenofovir; LPV: lopinavir.

*Advanced: WHO stage III/IV or CDC clinical stage C.

### Comparison of Kaplan-Meier and competing risk cumulative incidence

**Figure 1:**
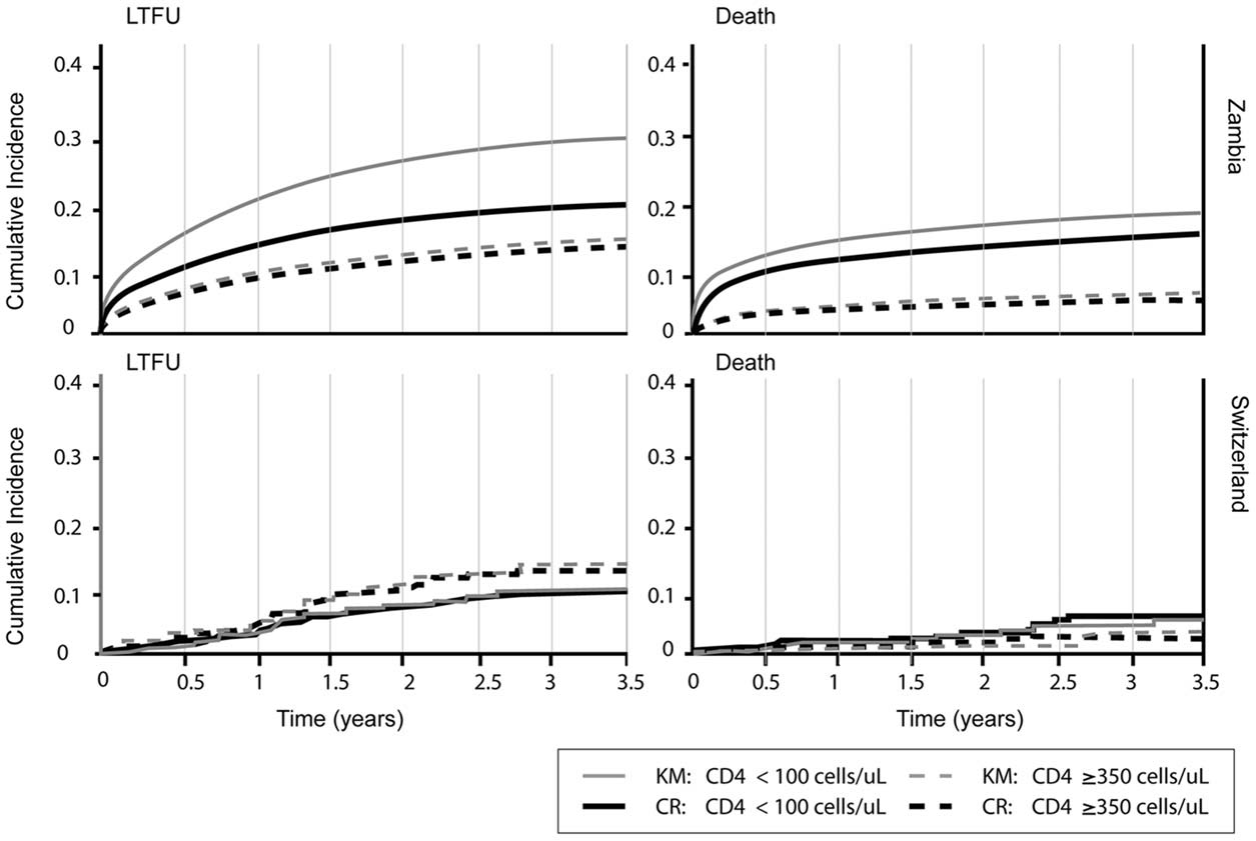
Standard Kaplan-Meier (KM) curves and cumulative incidence curves from competing risk (CR) analyses of loss to follow-up and death in the CIDRZ cohort in Zambia and the Swiss HIV Cohort Study.

Figure 1 shows the naïve Kaplan-Meier curves and the competing risk cumulative incidence. In the naïve Kaplan-Meier analysis the proportion of patients lost to follow-up after 3.5 years in the CIDRZ cohort was 29.3% among patients starting ART with CD4 cell counts <100 cells/µL and 15.4% among patients starting ART with counts ≥350 cells/µL. When the competing risk of death was taken into account, estimates of loss to follow-up were lower: 22.9% for ART patients with CD4 counts <100 cells/µL at treatment initiation and 13.6% for ART patients with CD4 counts ≥350 cells/µL at treatment initiation. In patients starting with CD4 counts <100 cells/µL the estimated risk of death at 3.5 years was 19.9% in the KM analysis and 16.3% in the competing risk analysis. In patients with CD4 counts ≥350 cells/µL it was 8.8% and 6.3% respectively. In the SHCS, the results from the Kaplan-Meier analysis and the competing risk approaches were similar irrespective of baseline CD4 cell count, both for loss to follow-up and for mortality. Mortality was below 10% at 3.5 years in both CD4 cell strata and the influence of the competing risk was negligible.

### Rates, cause-specific hazard ratios and subdistribution hazard ratios

Rates of loss to follow-up declined in Zambia from 211.6 per 1,000 person-years in the first 6 months of ART to 53.5 per 1,000 person-years thereafter. The corresponding rates of death were 174.3 and 26.3 per 1,000 person-years. In the SHCS loss to follow-up declined from 31.8 to 26.8 per 1,000 person-years and death rates from 17.5 to 7.4 per 1,000 person-years in the same period.

Table 2 shows the results from the standard Cox models for mortality and loss to follow-up and the competing risk subdistribution model for loss to follow-up for different CD4 strata. The results from the two models were consistent. In Zambia the risk of loss to follow-up and death increased with decreasing CD4 counts at the start of ART. In Switzerland there was a trend in the opposite direction, with patients with higher CD4 cell counts more likely to be lost to follow-up. The cause-specific hazard ratios for death also increased with decreasing CD4 cell count but the association was somewhat weaker than in Zambia and not statistically significant. Finally, the hazard ratios comparing Switzerland with Zambia were below 1 for all CD4 strata, with slightly lower cause-specific hazard ratios, particularly at lower CD4 cell counts (Table 3).

**Table 2:** Unadjusted and adjusted hazard ratios across categories of baseline CD4 cell count in Zambia and Switzerland.

CD4 cell count	Subdistribution model			Cause-specific Cox model					
(cells/µL)	Loss to follow-up			Loss to follow-up			Death		
	sHR	95% CI	P	HR	95% CI	P	HR	95% CI	P
**Zambia**									
*Crude analysis*			<0.0001			<0.0001			<0.0001
<100	1.76	1.64–1.89		1.92	1.79–2.06		2.94	2.66–3.26	
100–199	1.15	1.06–1.24		1.17	1.08–1.26		1.52	1.36–1.69	
200–349	1.07	0.99–1.16		1.08	1.00–1.17		1.10	0.98–1.23	
≥350	1			1			1		
*Adjusted analysis* *			<0.0001			<0.0001			<0.0001
<100	1.66	1.54–1.77		1.84	1.71–1.97		2.69	2.43–2.97	
100–199	1.16	1.08–1.25		1.19	1.11–1.29		1.58	1.42–1.76	
200–349	1.07	0.99–1.16		1.08	1.00–1.17		1.12	0.99–1.25	
≥350	1			1			1		
**Switzerland**									
*Crude analysis*			0.38			0.40			0.37
<100	0.61	0.33–1.13		0.62	0.33–1.15		2.59	0.81–8.28	
100–199	0.92	0.54–1.55		0.94	0.55–1.59		2.04	0.63–6.64	
200–349	0.98	0.61–1.57		0.98	0.61–1.58		1.50	0.47–4.79	
≥350	1			1			1		
*Adjusted analysis* *			0.24			0.24			0.71
<100	0.57	0.30–1.11		0.57	0.30–1.12		1.96	0.58–6.63	
100–199	1.02	0.60–1.73		1.03	0.61–1.76		1.70	0.52–5.55	
200–349	1.07	0.66–1.73		1.07	0.66–1.74		1.36	0.42–4.35	
≥350	1			1			1		

Results for loss to follow-up from the subdistribution competing risk model and for loss to follow-up and death from standard Cox models are shown.

HR: hazard ratio; sHR: subdistribution hazard ratio; CI: confidence interval.

*Adjusted for age, gender, disease stage and body mass index.

**Table 3:** Unadjusted and adjusted hazard ratios for loss to follow-up from competing risk models and cause-specific Cox models comparing the Swiss HIV Cohort Study with the CIDRZ cohort in Zambia.

	Subdistribution model			Cause-specific Cox model		
	sHR	95% CI	P	HR	95% CI	P
***Crude***			<0.0001			<0.0001
<100	0.31	0.26–0.38		0.27	0.23–0.33	
100–199	0.48	0.40–0.58		0.44	0.37–0.54	
200–349	0.51	0.43–0.62		0.48	0.40–0.58	
≥350	0.55	0.45–0.67		0.52	0.43–0.63	
***Adjusted*** *			<0.0001			<0.0001
<100	0.30	0.18–0.48		0.24	0.16–0.42	
100–199	0.67	0.46–0.97		0.64	0.44–0.93	
200–349	0.69	0.51–0.93		0.68	0.51–0.92	
≥350	0.76	0.52–1.12		0.75	0.51–1.10	

HR: hazard ratio; sHR: subdistribution hazard ratio; CI: confidence interval.

*Adjusted for age, gender, disease stage and body mass index.

## Discussion

We compared outcomes in ART programme from Zambia and Switzerland to illustrate the importance of death as a competing risk when estimating loss to follow-up. Standard Kaplan-Meier analyses that ignored the competing risk of death substantially overestimated the cumulative incidence of loss to follow-up in patients starting with low CD4 counts in Zambia. In contrast, there was little bias among populations experiencing lower mortality, including patients starting ART with high CD4 counts in Zambia and all patients in Switzerland. The results from the cause-specific Cox models and the more complex Fine and Gray model were comparable, both when analyzing the effect of CD4 count strata on the rate of loss to follow-up, and when comparing the Swiss with the Zambian cohort.

We estimated the cumulative incidence of developing the event of interest (i.e., loss to follow-up) in the presence of a competing risk (i.e., death). The cumulative incidence represents the probability that an individual will experience an event of interest by time (*t*). In contrast to the standard Kaplan-Meier approach, the cumulative incidence from the competing risk analysis depends not only on the number of patients who experienced an event, but also on the number of patients who did not experience a competing event. Similarly, we used two approaches to model the effect of covariates in the present of the competing risk. The cause-specific Cox model, in which competing causes are censored, is a reasonable and practical choice but is restricted to modelling instantaneous risk functions [28]–[30]. The Fine and Gray model makes use of the subdistribution hazard to model cumulative incidence and thus quantify the overall benefit or harm of an exposure, however, it is considerably more complex [25], [28]. Of note, the effect of a covariate on cumulative incidence will also depend on its effect on the competing risk. In other words, the effect of a covariate on the cause-specific hazard may be different from the corresponding effect on cumulative incidence [12], [13]. This was recently illustrated using the example of the competing risks of stopping first line ART or switching to second-line ART [28].

Competing risk analyses are still rare in the literature, compared to standard survival analyses. In September 2011, MEDLINE returned only 85 hits when searching titles and abstracts with free text words “competing risk analysis” or “competing risk analyses”, with about half of articles from oncology. In contrast, repeating the search with words “Kaplan-Meier” or “Cox regression” returned thousands of hits. A possible explanation for the poor adoption of competing risk modelling in medical research is the lack of dedicated routines in commonly used statistical software. This is now changing with routines available in several packages, such as in R and Stata version 11.

An important strength of our study was the analysis of a combined dataset, which allowed using the same definitions and coding of variables in the Zambian and Swiss cohorts. We could thus examine the risk of loss to follow-up and death across the same CD4 categories, while adjusting for a common set of confounding variables. The CIDRZ programme is typical of many sites involved in the scale-up of ART in resource-limited settings. The scale-up follows a public health approach and must cope with the realities of constrained health systems, including health care worker shortages and limited diagnostic capacity for co-morbidities and co-infections. Key program characteristics include the standardisation of first-line and second-line regimens, simplified clinical decision-making, and standardised clinical and laboratory monitoring [31]. The choice of regimens is determined primarily by cost and can include drugs that are no longer widely used in industrialized countries. In Switzerland, by contrast, doctors prescribe from the full range of available antiretroviral drugs, resistance testing is used to individually tailor drug regimens, and CD4 cell counts and viral load are monitored frequently [32].

We found that the determinants of loss to follow-up differed in Zambia and Switzerland. In the African setting, sicker patients were more likely to be lost to follow-up, confirming previous studies from resource-limited settings [33], [34]. In Switzerland, there was a (statistically non-significant) trend in the opposite direction, in line with results from the French Hospital Database on HIV, which found that a history of an AIDS-defining illness was associated with reduced loss to follow-up [35], and the UK CHIC [36] and Hospital of Bergamo cohorts [37] where loss to follow-up was associated with a higher CD4 cell count.

We sought to illustrate the importance of competing risks when investigating the association between CD4 cell counts and loss to follow-up in Zambia and Switzerland. For example, we did not consider the different transmission groups in the SHCS but previous analyses showed that loss to follow-up was more common among current intravenous drug users (IDUs) compared to former IDUs and other risk groups [38]. Patients lost to follow-up are systematically traced in the SHCS to ascertain outcomes, but are not consistently traced in the CIDRZ cohort. Even when program resources are available, a significant proportion of patients cannot be contacted [20]. Reasons for follow-up losses are thus often unknown. Similarly, causes of deaths are not routinely collected in the CIDRZ cohort and no national death registries exist in Zambia to supplement this information. Furthermore, we used an intention-to-treat approach and thus ignored subsequent changes to treatment, including interruptions and terminations. An alternative approach would have been to account for treatment changes and time varying covariates by the use of inverse probability of treatment and censoring weights as in Cole et al [29]. Since drug interruptions and reasons for interruptions are not recorded systematically in the CIDRZ cohort we could not, however, use this approach.

The results obtained for mortality also deserve comment. It is important to note that cumulative mortality estimates from both the competing risk analysis and the Kaplan-Meier analysis may not be representative of all patients starting ART in the CIDRZ cohort (Figure 1). The competing risk analysis relates to patients remaining in care and thus estimates mortality during follow-up only: mortality in patients lost to follow up is not considered. The Kaplan-Meier method assumes that those lost to follow-up experience the same mortality as comparable patients remaining in care, ignoring the fact that in resource-limited settings mortality among patients lost is substantially higher than among patients remaining in care. In other words, censoring is highly “informative” [10]. Several methods have been proposed to correct for the under ascertainment of mortality due to loss to follow-up in these settings, including double sampling techniques, multiple imputations and nomogram-based adjustments [11], [33], [39]. In Switzerland the situation is different: loss to follow-up mainly reflects unknown transfers to other care givers, and deaths are ascertained by tracing patients and linking the data to routine mortality files.

In conclusion, our results demonstrate that death should be considered as a competing risk when estimating the cumulative incidence and determinants of loss to follow-up in settings where mortality is high. Continued efforts are needed to minimize loss to follow-up to improve patient outcomes in resource-limited settings.
